# Prediction of antipsychotics efficacy based on a polygenic risk score: a real-world cohort study

**DOI:** 10.3389/fphar.2024.1274442

**Published:** 2024-03-08

**Authors:** Marco De Pieri, Marco Ferrari, Giorgio Pistis, Franziska Gamma, Franca Marino, Armin Von Gunten, Philippe Conus, Marco Cosentino, Chin-Bin Eap

**Affiliations:** ^1^ Center for Research in Medical Pharmacology, Varese, Italy; ^2^ PhD Program in Clinical and Experimental Medicine and Medical Humanities, University of Insubria, Varese, Italy; ^3^ General Psychiatry Service, Hopitaux Universitaires de Genève, Geneva, Switzerland; ^4^ Unit of Pharmacogenetics and Clinical Psychopharmacology, Centre for Psychiatric Neuroscience, Department of Psychiatry, Lausanne University Hospital, University of Lausanne, Prilly, Switzerland; ^5^ Service of General Psychiatry, Department of Psychiatry, Lausanne University Hospital, University of Lausanne, Prilly, Switzerland; ^6^ Les Toises Psychiatry and Psychotherapy Center, Lausanne, Switzerland; ^7^ Service of Old Age Psychiatry, Department of Psychiatry, Lausanne University Hospital, University of Lausanne, Prilly, Switzerland; ^8^ School of Pharmaceutical Sciences, University of Geneva, University of Lausanne, Geneva, Switzerland; ^9^ Center for Research and Innovation in Clinical Pharmaceutical Sciences, University of Lausanne, Lausanne, Switzerland; ^10^ Institute of Pharmaceutical Sciences of Western Switzerland, University of Geneva, University of Lausanne, Lausanne, Switzerland

**Keywords:** antipsychotics, personalized medicine, GWAS, response to treatment, polygenic risk score, single nucleotide polimorphism (SNP)

## Abstract

**Background:** Response to antipsychotics is subject to a wide interindividual variability, due to genetic and non-genetic factors. Several single nucleotide polymorphisms (SNPs) have been associated with response to antipsychotics in genome-wide association studies (GWAS). Polygenic risk scores (PRS) are a powerful tool to aggregate into a single measure the small effects of multiple risk alleles.

**Materials and methods:** We studied the association between a PRS composed of SNPs associated with response to antipsychotics in GWAS studies (PRS_response_) in a real-world sample of patients (N = 460) with different diagnoses (schizophrenia spectrum, bipolar, depressive, neurocognitive, substance use disorders and miscellaneous). Two other PRSs composed of SNPs previously associated with risk of schizophrenia (PRS_schizophrenia1_ and PRS_schizophrenia2_) were also tested for their association with response to treatment.

**Results:** PRS_response_ was significantly associated with response to antipsychotics considering the whole cohort (OR = 1.14, CI = 1.03–1.26, *p* = 0.010), the subgroup of patients with schizophrenia, schizoaffective disorder or bipolar disorder (OR = 1.18, CI = 1.02–1.37, *p* = 0.022, N = 235), with schizophrenia or schizoaffective disorder (OR = 1.24, CI = 1.04–1.47, *p* = 0.01, N = 176) and with schizophrenia (OR = 1.27, CI = 1.04–1.55, *p* = 0.01, N = 149). Sensitivity and specificity were sub-optimal (schizophrenia 62%, 61%; schizophrenia spectrum 56%, 55%; schizophrenia spectrum plus bipolar disorder 60%, 56%; all patients 63%, 58%, respectively). PRS_schizophrenia1_ and PRS_schizophrenia2_ were not significantly associated with response to treatment.

**Conclusion:** PRS_response_ defined from GWAS studies is significantly associated with response to antipsychotics in a real-world cohort; however, the results of the sensitivity-specificity analysis preclude its use as a predictive tool in clinical practice.

## Introduction

Antipsychotics represent the mainstay in the treatment of schizophrenia and schizophrenia spectrum disorders and are among the principal therapeutic options for bipolar disorder (National institute for Heath and Care excellence NICE, 2014; Taylor et al., 2018; Yatham et al., 2018). Some antipsychotics can also be used as add-ons for treatment resistant unipolar major depression (Cantù et al., 2021) and many are extensively prescribed off-label for anxiety disorders (Pies, 2009; Hershenberg et al., 2014; Pignon et al., 2017), personality disorders (Rosenbluth and Sinyor, 2012; Wasylyshen and Williams, 2016; Yadav, 2020) and organic mental disorders (Elie and Rej, 2018). In the treatment of psychosis, response rates range from 47% for individuals who have received prior treatment to 66% for antipsychotic-naive individuals (Haddad and Correll, 2018), while 1-year discontinuation rates may be as high as 74% due to poor tolerability and/or lack of efficacy (Lieberman et al., 2005). Inter-individual variability in efficacy and side effects of antipsychotics is large and unpredictable, with several contributing factors, including nongenetic (e.g., lack of compliance, variability in pharmacokinetics, drug-drug interactions, environmental factors), genetic and epigenetic factors (Mackenzie et al., 2010; Pouget et al., 2014).

Among genetic factors, single nucleotide polymorphisms (SNPs) are relevant, contributing from 25% up to 50% of inappropriate drug responses, both in terms of efficacy and side effects (Allen and Bishop, 2019; Martin et al., 2019). Individual SNPs can be used according to a candidate gene approach to estimate their influence on drug response. More interestingly, they can be used together to build a polygenic risk score (PRS), combining multiple SNPs previously found to be associated to drug response in genome-wide association studies (GWAS), integrating into a single measure the sum of genetic influences (Martin et al., 2019). Of note, many GWAS have been published, aiming at elucidating the relationship between genetics and response to antipsychotic treatment, with the most relevant being systematically reviewed recently (Allen and Bishop, 2019; Koromina et al., 2020).

Thus, two studies managed to establish a positive relationship between response to treatment and a PRS (Zhang et al., 2019; Chen et al., 2020), while another did not (Santoro et al., 2018). Other studies focused on the relationship between a PRS and treatment-resistant schizophrenia (i.e., the clinical condition defined as the persistence of symptoms despite ≥2 trials of antipsychotic medications of adequate dose and duration with documented adherence (Demjaha et al., 2017)). Results were contradictory as well, since two studies established an association (Werner et al., 2020; Pardiñas et al., 2022) but another did not (Wimberley et al., 2017).

Studies including only highly selected and controlled patients determines a lack of generalizability to real-world clinical practice; for this reason, studies with a real-world design are becoming increasingly popular (Sherman et al., 2016).

The hypothesis of the study relies on the important genetic component in schizophrenia etiology and response to treatment, and on their polygenic nature; accordingly, we expected that a polygenic risk score could account for a significant proportion of variability in the individual response to antipsychotics.

The objective of the present study was to evaluate if a PRS, composed of 11SNPs previously associated with response to antipsychotic medications in GWAS studies (Allen and Bishop, 2019; Koromina et al., 2020), is associated with response to antipsychotic treatment in a real-world cohort (Allen and Bishop, 2019; Koromina et al., 2020).

## Methods

### Sample description

Four-hundred-and-sixty patients of European descent treated with antipsychotic(s) in the psychiatric service of the Lausanne University hospital as outpatients, inpatients or both, from 16.12.2006 to 09.09.2020, and with extensive genetic data available were selected in the cohort study. They are part of the PsyMetab study, an ongoing longitudinal cohort of over 3,000 psychiatric patients taking psychotropic medication, focusing on risks of weight gain and metabolic problems (PsyMetab, 2021; Sjaarda et al., 2023). Selected patients for the present study were the ones treated with an antipsychotic as primary therapy (see Table 1), combined in some cases with other antipsychotic(s), mood stabilizer(s), antidepressant(s) and/or benzodiazepine(s); 71% of the patients received an antipsychotic monotherapy.

**TABLE 1:** Prescribed antipsychotics.

	Median dose and range (mg/day)	N (total 460)
Amisulpride	400; 100–800	38
Aripiprazole	10; 2.5–40	93
Clozapine	200; 12.5–600	32
Haloperidol	5; 1–20	10
Lurasidone	40: 20–120	13
Levomepromazine	25	1
Olanzapine	10; 2.5–30	55
Paliperidone (oral)	6; 3–9	4
Paliperidone (long acting)	2.67	2
Quetiapine	400; 100–1000	116
Risperidone (oral)	4; 0.5–9	91
Risperidone (long acting)	3.57	1
Tiapride	400	1
Zuclopentixol	30; 10–30	3

Median dose and range and number of patients for each drug are indicated. Patients taking less than 100 mg of quetiapine were not included. Patients can receive more than one antipsychotic, only the one indicated as the primary treatment is considered. Daily dosage for long acting drugs was calculated dividing the dose by the interval of administration (i.e. 50 mg/14 days for risperidone long acting, 75 mg/28 days for paliperidone long acting); N = Number of patients.

Demographic covariates (i.e., sex, age and ethnicity) as well as history of treatment (e.g., psychotropic treatment duration) were obtained from medical files. Psychiatric clinical status was evaluated through Clinical Global Impressions-Severity (CGI-S) and Clinical Global Impressions-Improvement (CGI-I) rating scales, obtained through the PsyMetab database. Response to treatment was based on CGI-I and CGI-S scores and/or on the need for dose increases, a switch to another antipsychotic or mood stabilizer, an add-on and/or discontinuation of the treatment according to criteria described in Table 2. The clinical evaluation for each participant was performed for the first year after inclusion in the PsyMetab cohort.

**TABLE 2:** Criteria for the definition of responder and non-responder to treatment.

Criteria for response
1. Any positive variation of CGI-S of >= 2 points
2. Any CGI-S = 1
3. CGI-I <= 2
4. Duration of treatment >= 90 days
5. No other antipsychotics or mood stabilizers added
6. No dose increase after 90 days of treatment

Definition of responder (any of the 4 choices below)
1. Criteria 1 satisfied
2. Criteria 2 satisfied if not in contrast with criteria 1
3. Criteria 3 satisfied if not in contrast with criteria 1 and 2
4. Criteria 4 + 5 + 6 satisfied if not in contrast with criteria 1, 2 and 3

Criteria for non-response
1. Maximum positive variation of CGI-S< 2 points
2. CGI-I >2 at any observations
3. Beginning of a new follow up with a different antipsychotic, 75–105 days after withdrawal of the AP under study. The switch is not due to side effects of the discontinued medication
4. An antipsychotics or mood stabilizers added after beginning of the antipsychotic under study

Definition of non-responder (any of the 3 choices below)
1. Criteria 1 satisfied
2. Criteria 2 satisfied if not in contrast with criteria 1
3. Criteria 3 or 4 satisfied if not in contrast with criteria 1 and 2

Responder status was considered as unknown in cases not described above and such patients were not included in the analysis. For each participant, a period of 1 year after the inclusion in PsyMetab cohort was considered. Variation of CGI considered were at least 30 days apart. CGI-S = clinical global impression, severity of symptoms scale; CGI-I = clinical global impression, improvement scale.

Psychiatric diagnoses were made according to ICD-10 classification criteria. Diagnoses were proposed by a senior psychiatrist by means of a semi-structured interview, and/or by means of a prolonged longitudinal evaluation. The main diagnostic groups were [F20.0-F24.9] & [F28-F29]: psychotic disorders; [F25.0-F25.9]: schizoaffective disorders; [F30.0-F31.9]: bipolar disorders; [F32.00-F33.9]: depression; [F03.0-F03.9]: dementia; [F10.0-F19.99]: substance use disorder; miscellaneous. The PsyMetab study was approved by the Ethics Committee of the Canton of Vaud (CER-VD) Lausanne University Hospital (approval number 2017–01301); written informed consents, also for genetic analysis, were obtained from all the participants.

### SNP selection, genotyping and construction of PRSs

Genetic variants were determined by standard genotyping or imputation methods. DNA samples from all patients were genotyped using the *Global Screening Array (GSA) v2 with multiple disease option* processed on an iScan equipped platform (Illumina, San Diego, CA) at the iGE3 genomics platform of the University of Geneva (https://www.ige3.unige.ch/). Quality control methods were applied as follows: SNPs were excluded on the basis of low call rate (<99%), deviation from HW-equilibrium (p < 1 × 10^−6^), or low minor allele frequency (MAF<0.05). Sex mismatches or cryptic relatedness were also removed. All quality control steps were performed using PLINK (https://www.cog-genomics.org/plink/2.0/). Finally, all autosomal variants were submitted to the Michigan imputation server (https://imputationserver.sph.umich.edu/index.html#!). The server uses SHAPEIT2 (v2. r790) to phase data and imputation to the reference panel (1000G phase 1 version 3) was performed with Minimac 3.7.8.

Polygenic risk scores were built with the “standard weighted allele” method implemented in PRSice-2 (Chen et al., 2018). Linkage disequilibrium (LD) clumping was performed to retain only data for independent SNPs (r2 < 0.1, clump-p = 1, 250 kb windows).

The main PRS (PRS_response_) was built using 11 SNPs (see Table 3). The rationale was to combine SNPs previously associated with response to antipsychotics in GWAS studies, as from two recent reviews (Allen and Bishop, 2019; Koromina et al., 2020), and having a minor allele frequency in the European descent population >5%. We aimed to replicate findings from the source studies, testing each individual SNP for association with response, and to determine if the combination of SNPs in a PRS could improve the prediction of response to antipsychotics.

**TABLE 3:** SNPs linked to response to antipsychotic treatment at a GWAS significant level.

SNP	Gene	Risk allele	P	Efficacy measure	Antipsychotic	References
rs6688363	*ATP1A2*	T	1.6 × 10^−7^	CGI-S improvement	Risperidone	Clark et al. (2013)
rs10170310	*SPOPL*	G	1.0 × 10^−7^	PGI improvement	Quetiapine	
rs7395555	*intergenic*	C	2.0 × 10^−7^	CGI-S improvement	Risperidone	
rs711355	*TJP1*	T	2.3 × 10^−7^	PGI improvement	Risperidone	
rs17382202	*PDE4D*	T	4.2 × 10^−8^	PGI improvement	Olanzapine	
rs2980976	*TNFRSF11A*	A	3.0 × 10^−7^	CGI-S improvement	Risperidone	
rs1875705	*GRID2*	A	1.1 × 10^−8^	BPRS total score	Risperidone	Stevenson et al. (2016)
rs2133450	*GRM7*	C	4.3 × 10^−8^	PANSS positive score	Risperidone	Sacchetti et al. (2017)
rs10023464	*intergenic*	T	9 × 10^−66^	Response to Clozapine	Clozapine	Pardiñas et al. (2019)
rs7668556	*intergenic*	A	4 × 10^−13^	Response to Clozapine	Clozapine	
rs12767583	CYP2C19	T	1 × 10^−16^	Response to Clozapine	Clozapine	Smith et al. (2020)

CGI-S, clinical global impression, Severity of Symptoms scale; PGI, Patient’s Global Impression; PANSS, positive and negative syndrome scale; BPRS, brief psychiatric rating scale.

Four other SNPs obtained from these studies were ruled out for the following reasons: rs17390445 was not used because the risk allele for response to treatment was not available from the original author; rs11725502 and rs10023464 were in total linkage disequilibrium, as well as rs17742120, rs2164660 and rs17382202: rs11725502, rs17742120 and rs2164660 were therefore not included in the PRS.

In addition, 2 PRSs composed on SNPs related to the risk of schizophrenia were built. PRS_schizophrenia1_ used 94 SNPs from a 2014 study realized by the Psychiatric Genomic consortium (Schizophrenia Working Group of the Psychiatric Genomics Consortium, 2014); PRS_schizophrenia2_ used 313 SNPs from a novel study performed by the same group in 2022 (Trubetskoy et al., 2022).

The aim of using these two PRSs was to have a control to confirm the non-random nature of the results eventually obtained with PRS_response_, and to explore the hypothesis that SNPs conferring a risk of schizophrenia could also be linked to response to antipsychotic treatment. PRS_response_ had no SNP in common with PRS_schizophrenia1_ and PRS_schizophrenia2_.

Building PRS_response_, the effect of each SNP could not be weighted, since beta-coefficients for response to treatment were unknown; a dummy beta-value of 1 was used, and genotypes from each SNP were coded as 0, 1 or 2 according to the number of risk alleles for non-response to antipsychotic treatment (PRS_response_). For PRS_schizophrenia1_ and PRS_schizophrenia2_ the influence of each SNPs was weighted based on odds-ratios.

### Statistics

Ethnicity was assessed by the patient’s reported ethnicity and confirmed by genotyping using a principal component analysis with the EIGENSTRAT algorithm implemented in GCTA software. The majority of the variance was explained by the first two vectors and the European descent was arbitrarily selected when pca1<0.0025 and pca2>−0.0125, values which gave the highest concordance with the patient’s reported ethnicity. For further precision, principal components PC1, PC2, PC3, PC4 and PC5 were used in the statistical model described below.

Hardy-Weinberg Equilibrium (HWE) was determined for each polymorphism by a chi-square test. Descriptive analysis of quantitative data are presented as median and range unless otherwise specified whereas qualitative data are expressed as percentages. The chi-squared test or rank sum test were used for association studies within categorical data or non-parametric continuous variables, respectively. *p*-values equal to or less than 0.05 were considered statistically significant.

Influence of possible confounders on the variable of interest (response to treatment) were analyzed through a univariate analysis based on a linear regression model. We derived a list of prognostic factors for response to antipsychotic treatment which includes premorbid global functioning, education, age of onset, sex, psychiatric comorbidities (especially substance abuse), duration of untreated psychosis (DUP), duration of disease, baseline severity and response to treatment in the first 2 weeks (Chandola and Jenkinson, 2000; Carbon and Correll, 2014; Bozzatello et al., 2019). According to data available in our database we studied the possible effect of age (used as a proxy for age of onset and duration of untreated psychosis), duration of disease, sex, socioeconomic status (used as a proxy for premorbid global functioning and education), and of comorbid substance abuse. Socioeconomic status was obtained with a GPS-based classification system, as previously described (Takeuchi et al., 2019; Dubath et al., 2020). Other variables of interest such as duration of untreated psychosis and treatment response in the first 2 weeks were not available in our dataset.

Moreover, relapses of psychotic episodes are known to cause resistance to antipsychotic treatment (Ruopp et al., 2008; Taipale et al., 2022). In order to account for this possible confounder we evaluated the relationship between the number of admissions to a psychiatric unit and response to treatment in the cohort.

In univariate analysis, age was significantly associated with response to treatment (*p* < 0.004, data not shown) in the whole cohort, a result confirmed in multivariate analysis (data not shown). Other variables listed above showed no associations with response to treatment and were therefore not included in multivariate analysis. A logistic regression model linking each PRS (PRS_response_, PRS_schizophrenia1_ and PRS_schizophrenia2_) with response to treatment was performed, including the whole group and specific diagnostic subgroups. In the logistic regression model, PRS, age and the 5 principal genetic components (PC1-PC5) were used as predictor variables, and response to antipsychotics was the outcome variable. Sensitivity, specificity, positive predictive value (PPV) and negative predictive value (NPV) were calculated for PRS, predicting response to treatment. Cut-off values for the sensitivity-specificity analysis were chosen in order to maximize the Youden index (Wray et al., 2007).

## Results

### Population description

**FIGURE 1:**
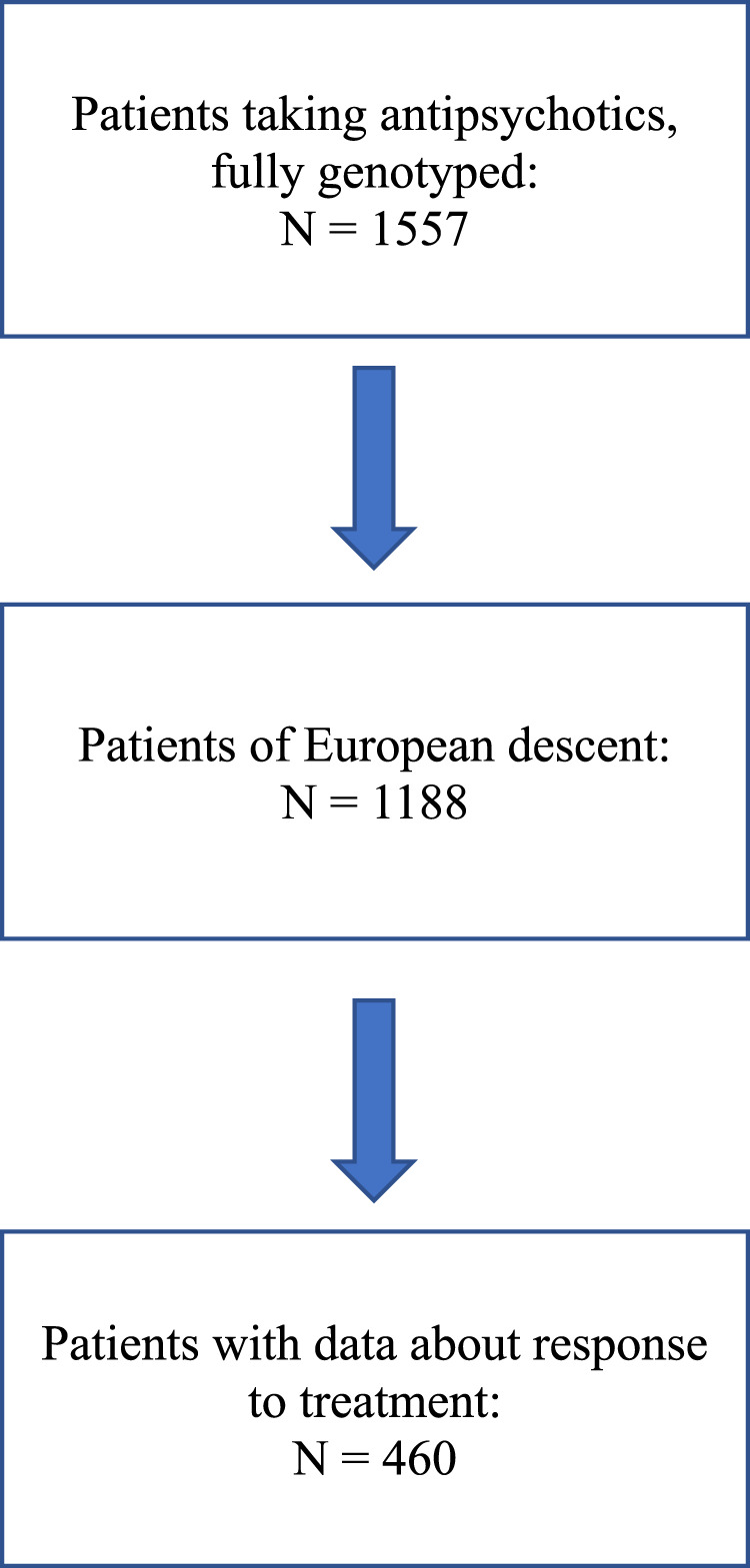
Flow chart of patients’ selection from PsyMetab cohort.

A flow chart describing patient selection is shown in Figure 1.

Table 4 presents the characteristics of the included patients, consisting of 460 individuals of european descent, of which 249 were responders and 211 non-responders to antipsychotic treatment. Non-responders were significantly older than responders (48.4 years vs. 42.2 years, *p* < 0.002), while other factors known to predict a worse response to antipsychotics, such as male gender, low socioeconomic status, longer duration of illness, higher baseline severity of the illness and substance abuse (Chandola and Jenkinson, 2000; Bozzatello et al., 2019; Dubath et al., 2020) and the number of admissions to a psychiatry unit were similarly distributed in both groups.

**TABLE 4:** Clinical features of the sample under study.

	Responders			Non responders			P
	*Median* *[* *IQR*]	*Range*	*N*	*Median* *[* *IQR*]	*Range*	*N*	
Age, years	37 [25|56]	13–90	249	46 [30|68.5]	12–90	211	0.002*
Gender (Female, %)	50.6			49.5			0.45^a^
Duration of disease, years	4 [1|11]	0–39		4 [1|11]	0–50		0.80*
Period of evaluation, days	250 [180|340]	160–365		245 [185|343]	148–365]		0.82*
Number of admissions in psychiatry	2 [1|3]	0–29		2 [1|4]	0–29		0.63*
Socio-economic status	57.7 [43.6|66.6]	13.9–81.3		59.6 [50|66.4]	19–79.4		0.20*
CGI-S at first observation	4 [2|5]	1–7		5 [4|5]	1–7		0.30*
Substance abuse (%)	16.5			16			0.51^a^

[IQR] interquartile range, expressed as 25th percentile|75th percentile; N number of patients; P *p*-value for the difference between responders and non-responders; * Wilcoxon rank-sum test.

^a^ Fisher exact test; CGI-S, clinical global impression, severity of symptoms scale. Significant *p*-values in bold.

### Association of individual SNPs with response to AP

The relationship between response to treatment and each individual SNP composing PRS_response_ was first considered. No statistically significant associations were found, both considering the whole cohort (see Table 5) and subgroups of patients (i.e., schizophrenia spectrum plus bipolar disorder, schizophrenia spectrum, schizophrenia, schizoaffective disorder, bipolar disorder, others; data not shown).

**TABLE 5:** Correlation between patient genotypes and response to antipsychotics.

GENE	SNP	Genotype	Responders (N)	Non-responders (N)	P	Odds ratio (95% C.I.)
*ATP1A2*	rs6688363	C/C	157	146	0,17^a^	0.84 (0.6–1.2)
		C/T	76	56		
		T/T	16	10		
*SPOPL*	rs10170310	A/A	184	145	*0,08* ^a^	1.3 (0.9–1.9)
		A/G	62	58		
		G/G	3	9		
*GRM7*	rs2133450	A/A	62	64	*0,06* ^a^	0.6 (0.5–1.5)
		A/C	118	105		
		C/C	69	43		
*Intergenic*	rs10023464	C/C	207	188	*0,07* ^a^	0.6 (0.4–1.1)
		C/T	38	23		
		T/T	4	1		
*Intergenic*	rs7668556	T/T	78	80	*0,06* ^a^	0.75 (0.5–1.1)
		T/A	120	101		
		A/A	51	31		
*GRID2*	rs1875705	A/A	82	61	0,35^a^	(0.8–1.8)
		A/G	118	105		
		G/G	49	46		
*PDE4D*	rs17382202	C/C	160	134	0,89^a^	0.9 (0.6–1.4)
		C/T	80	71		
		T/T	9	7		
*CYP2C19*	rs12767583	C/C	171	155	0,17^a^	0.8 (0.5–1.2)
		C/T	69	54		
		T/T	9	3		
*Intergenic*	rs7395555	G/G	156	145	0,20^b^	0.8 (0.5–1.1)
		G/C	88	59		
		C/C	5	8		
*TJP1*	rs711355	C/C	96	84	0,85^b^	0.9 (0.6–1.4)
		C/T	114	94		
		T/T	39	34		
*TNFRSF11A*	rs2980976	G/G	175	151	0,84^b^	0.9 (0.6–1.4)
		G/A	69	55		
		A/A	5	6		

ATP1A2, Sodium/potassium-transporting ATPase subunit alpha-2; SPOPL, Speckle Type BTB/POZ Protein Like; GRM7, Metabotropic glutamate receptor 7; GRID2, Glutamate Ionotropic Receptor Delta Type Subunit 2; PDE4D, Phosphodiesterase 4D; CYP2C19, Cytochrome P450 Family 2 Subfamily C Member 19; TJP1, Tight Junction Protein 1; TNFRSF11A, TNF Receptor Superfamily Member 11A. P *p*-value, trends towards significance are in italics.

^a^ = χ2-test for trend.

^b^ = Fisher exact test.

### Association of PRS_response_, PRS_schizophrenia1_ and PRS_schizophrenia2_ with response to APs

PRS_response_ was significantly associated with response to antipsychotic treatment considering all patients without differentiation based on diagnostic category (OR = 1.14, CI = 1.03–1.26, N = 460, see Table 6), but also in the group of patients with schizophrenia, schizoaffective disorder or bipolar disorder (OR = 1.18, CI = 1.02–1.37, N = 235) and in the group of patients with schizophrenia and schizoaffective disorder (OR = 1.27, CI = 1.04–1.47, N = 176). When considering single diagnosis, a significant association was observed for schizophrenia (OR = 1.27, CI = 1.04–1.55, N = 149) but not for schizoaffective disorder (OR = 1.78, CI = 0.91–3.52, N = 27), bipolar disorder (OR = 1.00, CI = 0.71–1.41, N = 59) or another diagnosis of minor interest for antipsychotic prescription (OR = 0.98, CI = 0.76–1.26, N = 96), each represented by few patients (depression, N = 42; neurocognitive disorders, N = 17; substance abuse disorder, N = 9; others, N = 28). The most relevant results for PRS predicting the response to treatment were as follows: for the whole cohort of patients sensitivity was 63%, specificity 58%, PPV 64%, NPV 57%; for the group of patients with schizophrenia sensitivity was 62%, specificity 61%, PPV 66%, NPV 57%.

**TABLE 6:** PRS_response_ values in responders and non-responders and logistic multivariate regression analysis between PRS_response_ and response to treatment.

	N	PRS_response_ mean ± SD (range)		Or (95%C.I.)	P	Sensitivity (%)	Specificity (%)	PPV (%)	NPV (%)	Cutoff
		*Responders*	*Non-responders*							
**All patients**	460	6.15 ± 1.96 (1.99–11.91)	5.69 ± 1.92 (0.098–11.96)	1.14 (1.03–1.26)	**0.01**	63	58	64	57	5.66
**SCZ, SA, BD**	235	6.19 ± 1.79 (1.99–10.86)	5.60 ± 1.94 (0.098–11.96)	1.18 (1.02–1.37)	**0.02**	60	56	62	53	5.58
**SCZ, SA**	176	6.26 ± 1.77 (2.97–10.86)	5.52 ± 2.06 (0.098–11.96)	1.24 (1.04–1.47)	**0.01**	56	55	59	52	5.02
**SCZ**	149	6.13 ± 1.76 (2.97–10.86)	5.34 ± 1.90 (1.96–11.96)	1.27 (1.04–1.55)	**0.01**	62	61	66	57	5.02
**SA**	27	7.13 ± 1.67 (5.01–9.89)	6.32 ± 2.60 (0.098–9.03)	1.78 (0.91–3.52)	0.09	83	87	83	87	5.00
**BD**	59	6.02 ± 1.84 (2.00–10.75)	5.91 ± 1.47 (3.94–8.97)	1.00 (0.71–1.41)	0.98	86	29	64	58	5.33
**Others**	96	6.00 ± 2.08 (2.00–10.95)	5.96 ± 1.86 (1.97–9.93)	0.98 (0.76–1.26)	0.87	66	77	74	70	6.97

Results corrected for age PC1, PC2, PC3, PC4, PC5. SCZ, schizophrenia; SA, schizoaffective disorder; BD, bipolar disorder; Others include neurocognitive disorder, substance abuse disorder, depression, miscellaneous; N, number of patients; OR, odds ratio; 95%CI: 95% confidence interval; P, *p*-value, statistically significant results in bold; PC, genetic principal components; PPV, positive predictive value; NPV, negative predictive value; cutoff, empirical optimal cut-off value to separate responders and non-responders, calculated according to the Youden’s method.

**FIGURE 2:**
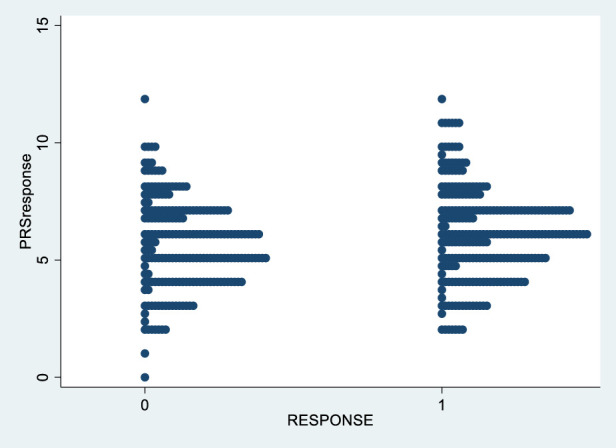
PRS_response_ in responder and non-responder to treatment.

Figure 2 shows the distribution of PRS_response_ in responders and non-responders to treatment.

Using a logistic multivariate regression controlling for age, PRS_schizophrenia1_ was not associated with response to treatment either in the whole group, nor in any of the abovementioned diagnoses (see Table 7).

**TABLE 7:** PRS_schizophrenia1_ values in responders and non-responders and logistic multivariate regression analysis between PRS_schizophrenia1_ and response to treatment.

	N	PRS mean ± SD (range)		OR (95% CI)	P	Sensitivity (%)	Specificity (%)	PPV (%)	NPV (%)	Cutoff
		Responders	Non-Responders							
All patients	460	−1.03 ± 0.42 (−1.93/0.20)	−1.05 ± 0.39 (−2.23/0.14)	1.06 (0.66–1.69)	0.80	58	56	61	53	−0.56
SCZ, SA, BD	235	−1.01 ± 0.41 (−1.90/0.20)	−1.06 ± 0.36 (−2.23/−0.28)	1.28 (0.65–2.54)	0.47	60	57	63	54	−1.04
SCZ, SA	176	−0.98 ± 0.41 (−1.93/0.20)	−1.07 ± 0.35 (−1.99/−0.28)	1.70 (0.76–3.79)	0.19	62	62	65	59	−1.04
SCZ	149	−0.99 ± 0.38 (−1.93/−0.25)	−1.11 ± 0.34 (−1.99/−0.28)	2.28 (0.87/5.97)	0.09	61	63	67	57	−1.04
SA	27	−0.97 ± 0.61 (−1.77/0.20)	−0.96 ± 0.38 (−1.60/−0.34)	1.18 (0.097–14.30)	0.90	67	93	89	78	−1.08
BD	59	−1.07 ± 0.41 (−1.75/−0.05)	−0.98 ± 0.39 (−2.23/−0.35)	0.51 (0.12–2.08)	0.35	57	58	67	48	−0.57
Others	96	−1.11 ± 0.39 (−1.76/−0.20)	−1.01 ± 0.40 (−2.02/0.14)	0.71 (0.21–2.37)	0.57	68	76	73	71	−0.33

Results corrected for age, PC1, PC2, PC3, PC4, PC5. SCZ schizophrenia; SA, schizoaffective disorder; BD bipolar disorder; others include neurocognitive disorder, substance abuse disorder, depression, miscellaneous; N, number of patients; OR, odds ratio; 95% CI: 95 confidence interval; P, *p*-value; PC genetic principal components; PPV, positive predictive value; NPV, negative predictive value; cutoff, empirical optimal cut-off value to separate responders and non-responders, calculated according to the Youden’s method.

Similarly, PRS_schizophrenia2_ showed no relationship with response to antipsychotics, in any diagnostic group (see Table 8).

**TABLE 8:** PRS_schizophrenia2_ values in responders and non-responders and logistic multivariate regression analysis between PRS_schizophrenia_ and response to treatment.

	N	PRS mean ± SD (range)		OR (95% CI)	P	Sensitivity (%)	Specificity (%)	PPV (%)	NPV (%)	Cutoff
		Responders	Non-Responders							
All patients	460	−0.37 ± 0.61 (−2.14—1.21)	−0.33 ± 0.55 (−1.65—1.05)	0.82 (0.59–1.14)	0.24	61	55	62	54	−3.5
SCZ, SA, BD	235	−0.34 ± 0.59 (−2.14/1.08)	−0.31 ± 0.58 (−1.65/1.05)	1.04 (0.73–1.48)	0.82	60	57	63	54	−2.65
SCZ, SA	176	−0.36 ± 0.59 (−2.14/1.08)	−0.30 ± 0.61 (−1.65/1.04)	0.88 (0.53–1.46)	0.62	66	62	67	61	−0.90
SCZ	149	−0.34 ± 0.61 (−2.14/1.08)	−0.32 ± 0.57 (−1.54/1.05)	0.98 (0.55–1.70)	0.93	59	57	62	53	−3.01
SA	27	−0.41 ± 0.45 (−1.13/0.24)	−0.24 ± 0.80 (−1.65/0.83)	0.80 (0.16–0.94)	0.78	67	93	89	78	−1.32
BD	59	−0.31 ± 0.59 (−1.64/0.52)	−0.31 ± 0.46 (−1.23/0.56)	1.11 (0.35–1.00)	0.86	57	50	62	44	−0.010
Others	96	−0.36 ± 0.56 (−2.09/0.89)	−0.33 ± 0.54 (−1.52/0.88)	0.61 (0.25–1.47)	0.27	68	71	70	70	−3.47

Results corrected for age, PC1, PC2, PC3, PC4, PC5. SCZ schizophrenia; SA, schizoaffective disorder; BD bipolar disorder; others include neurocognitive disorder, substance abuse disorder, depression, miscellaneous; N, number of patients; OR, odds ratio; 95% CI: 95 confidence interval; P, *p*-value; PC genetic principal components; PPV, positive predictive value; NPV, negative predictive value; cutoff, empirical optimal cut-off value to separate responders and non-responders, calculated according to the Youden’s method.

## Discussion

The present study aimed to determine whether PRS_response_, integrating SNPs previously associated with response to antipsychotics, predict the likelihood of response to antipsychotics in a naturalistic cohort including multiple diagnostic categories. As a secondary aim, we also evaluated the relationship of response to antipsychotics with PRS_schizophrenia1_ and PRS_schizophrenia2_, PRS_response_ was significantly associated with response to antipsychotics when considering the whole cohort and 3 subgroups: one composed of patients with schizophrenia; one of patients with schizophrenia or schizoaffective disorder (i.e., the schizophrenia spectrum); and one of patients with schizophrenia, schizoaffective disorder or bipolar disorder. No association was found in subgroups of patients with schizoaffective disorder, with bipolar disorder, with depression, or with another diagnosis, which could be also due to the smaller number of patients.

As indicated by *p*-values when considering diagnostic sub-groups, the statistical significance in the whole cohort and in multiple-diagnoses subgroups seemed driven by patients with schizophrenia, while relevant relationships were absent for the other diagnoses. However, odds-ratio were similar among different diagnostic groups, and the sample sizes were smaller for diagnosis other than schizophrenia; as a consequence, due to low statistical power, the diagnosis-specific character of PRS_response_ remains to be determined.

Subsequent analysis for all diagnostic groups revealed modest sensitivity, specificity, PPV and NPV; these findings indicate that PRS_response_ cannot be translated into a predictive tool for clinical practice. One exception is represented by the group with schizoaffective patients, in which we values of sensitivity and specificity were notheworthy. However, the corresponding *p*-value and odds ratios were not significant, cancelling the relevance of the sensitivity-specificity values.

Of note, when considering individually each single SNP composing PRS_response_, no statistically significant association was found with response to treatment, indicating that PRS_response_ has a stronger relationship with response to treatment than single SNPs.

No significant associations with response to treatment were found for both PRS_schizophrenia1,_ and PRS_schizophrenia2_, indicating that, at least in the present cohort, genes involved in response to antipsychotic treatment are stronger determinants of response than genes involved in risk of schizophrenia. We explored the relationship of PRS_schizophrenia1_ and PRS_schizophrenia2_ with response to treatment in order to embrace a different approach, not considering candidate genes but instead the summary statistics of a large GWAS to build the polygenic risk score. Present results are in contrast with a previous study that discovered a significant relationship between PRS_schizophrenia_ and response to antipsychotics (Zhang et al., 2019). However, many differences in methods can explain the contrasting outcome: the latter study included a higher number of patients (N = 510), all patients were diagnosed with a first episode of schizophrenia; all 108 loci from the seminal study of the Psychiatric Genomic Consortium (Schizophrenia Working Group of the Psychiatric Genomics Consortium, 2014) were used to build PRS_schizophrenia_, while 14 were missing in our dataset; PRS_schizophrenia_ was the weighted sum of SNPs; a different definition of response to treatment was used; first-generation antipsychotics were used more often than in the present cohort; and multiple ethnicities were considered, while the present study focused on patients of European descent.

Two other studies examined the relationship between PRS_schizophrenia_ and treatment-resistant schizophrenia, with one (Werner et al., 2020) positive and one negative result (Wimberley et al., 2017). Also, these studies differed from the present one in several ways (number of patients, rating scale used to evaluate response, duration of the antipsychotic trial, and the weighting of SNPs included in the PRS).

Three other studies on the relationship between a PRS and response to antipsychotics have been published, in which PRS was based on a case-control GWAS design, using all loci available from a large cohort’s summary statistics. Therefore, the primary objective of GWAS analysis performed in these studies was to build a PRS and not to find single loci significantly associated with response to antipsychotics, which was the case of the source studies used to build PRS_response_ in the present one. The underlying differences in methodology leaded to different SNP selection (in particular, *p*-value thresholds for statistical significance), with a lower number of SNPs included in the PRSs in the present study. A case-control study of patients with first-episode psychosis treated with risperidone failed to establish a significant association between a PRS and response (evaluated with the positive and negative syndrome scale), even though significant associations were found with some secondary outcomes (Santoro et al., 2018). A second study classified schizophrenia into distinct subtypes with different dimensions of genetic risk, using an ensemble of 10 GWAS-based PRSs for comorbid traits. The analysis produced 5 clusters of patients differing for positive, negative and cognitive symptom improvement (Chen et al., 2020). A third study found a significant association between a PRS and treatment-resistant schizophrenia, even if the proportion of variance explained was low (Pardiñas et al., 2022). Differences of methodology (e.g., construction of the PRS) and clinical characteristics (e.g., number of patients, age, diagnosis, number of psychotic episodes, type of antipsychotics) between the present and the abovementioned studies (Pardiñas et al., 2019; Chen et al., 2020; Pardiñas et al., 2022) make it difficult to compare results. However, no PRS_response_ has a sufficient predictive power to be used in clinical practice, genetic factors explaining the variability in response to antipsychotics s only to a limited extent.

Of note, even if the selection of SNPs in the present study is based on GWAS studies, which is a probabilistic approach making no assumptions on the function of selected genetic variants, it is noteworthy that most of the SNPs are in genes linked to the pathogenesis of psychosis and/or to response to antipsychotics. Only rs10023464, rs7668556 and rs7395555 are located in intergenic regions, with no known regulatory role (Clark et al., 2013; Ensembl, 2024). Rs6688363 is in *ATP1A2* (Seabra et al., 2020), coding for a Na/K ATPase expressed at multiple levels in the central nervous system (Ensembl, 2024), including the basal ganglia (Yue et al., 2009) and, therefore, possibly influencing the neuronal electrical activity. Rs10170310 is in *SPOPL* (Ensembl, 2024), involved in the Hedgehog pathway and in the ubiquitin-proteasome system (Seabra et al., 2020), possibly affected by antipsychotic administration (Sacchetti et al., 2017). Rs2133450 is in the glutamate metabotropic receptor *GRM7* (Stevenson et al., 2016) and rs1875705 in the NMDA receptor subunit *GRID2* (Stahl, 2018), likely modulating glutamatergic neurotransmission, whose dysfunction contributes to schizophrenia (Schwartz et al., 2012; Smith et al., 2020). Rs17382202 is in the phosphodiesterase 4D (PDE4D) gene (Ensembl, 2024), encoding for a key enzyme in nitric oxide neurotransmission (Yue et al., 2009), whose role in the pathogenesis of schizophrenia is under investigation. Rs711355 is in the gene coding for TJP1, a protein involved in the formation of brain tight junctions (Ensembl, 2024). Rs12767583 (Leucht and Engel, 2006) is in the coding region of CYP2C19 (Jürgens et al., 2020), and can therefore influence the response to treatment modulating the availability of its substrate clozapine (Najjar et al., 2013). Rs2980976 is located on TNFRSF1A (Ensembl, 2024), coding for a protein belonging to the family of TNF-alfa receptors, expressed in the brain and playing a role in the pathway of NFkB (Yue et al., 2009), a protein involved in immune response, neuroinflammation being an established feature of many mental disorders, including schizophrenia and bipolar disorder (Leucht et al., 2007).

The present study has several limitations. The number of patients is modest, and the antipsychotics under study are heterogeneous for mechanisms of actions and doses prescribed. Included patients had a hetegenous history of medication, from first antipsychotic treatment to multiple treatments and treatment resistance. However, a careful preliminary univariate statistical analysis was conducted to assess the influence of confounders on response to treatment. Only patients of European descent were included and results cannot be extrapolated to other ethnicities. Diagnostic categories evaluated were heterogeneous, including not only schizophrenia and bipolar disorder but also other categories in which antipsychotics are often prescribed off-label. Even if this choice introduced further variability, it aimed to expand the knowledge on antipsychotics pharmacogenomics beyond schizophrenia and bipolar disorder. Moreover, to address this possible limitations, the analyses were conducted not only at the whole cohort level but also in single-diagnosis subgroups. Major limitations of this study concern the criteria for response to antipsychotics, not based on the standard use of Positive and Negative Syndrome Scale (PANSS) or of Brief Psychiatric Rating Scale (BPRS) (Leucht and Engel, 2006). However, CGI demonstrated a high level of concordance with PANSS and BPRS in evaluating psychosis severity and antipsychotic treatment outcome and proved to be an effective tool to these ends (LEON et al., 1993; Rabinowitz et al., 2006; Rabinowitz et al., 2010). In addition, compared to PANSS and BPRS, CGI offers the advantage of being applicable to all mental disorders, fitting the present study design. Lastly, CGI measures were shown to have good internal consistency and concurrent validity (Purgato and Barbui, 2013). Indirect indicators of treatment efficacy (i.e., switch to a different antipsychotic, add-on of a novel antipsychotic or mood stabilizer, long duration of an antipsychotic treatment without dose increase) were used when CGI was not available. Even if their use as a criteria for response is to our knowledge unprecedented, according to our clinical experience, they are strongly related to efficacy of treatment, especially when side effects as a cause for treatment discontinuation have been ruled out, as in the present study.

A possible issue related to response criteria was the fact that response to treatment was a dichotomic outcome. Dichotomizing a variable as response to treatment poses several issues such as an underestimate of the extent of variation in outcome between groups, a loss of statistical power and an increase in the risk of a type 1 error. Moreover, the definition of a cut-point could be arbitrary, and produces large differences in proportions. On another side, keeping data continuous forces to express results in terms of mean and standard deviations, while in clinical practice to label individuals as having or not having an outcome makes the interpretation of results easier. Moreover, dichotomization allows to use measures such as risk difference, relative risk and odds ratio and it simplifies statistical analysis (Altman and Royston, 2006; Leucht and Lasser, 2006). As a matter of fact, gold standard criteria to determine response to antipsychotics revolve around a binary definition of response as a 50% reduction in a rating scale score such as BPRS or PANSS (Leucht et al., 2007; Cole et al., 2019). In our criteria for response a variation in the score of CGI-S and CGI-I was used in a way mirroring the change in BPRS or PANSS indicated above (LEON et al., 1993; Rabinowitz et al., 2006).

Age range was wide, and represented a relevant source of heterogeneity. In fact, an earlier onset of the disease is a negative prognostic factor for schizophrenia and for response to treatment (Chandola and Jenkinson, 2000; Carbon and Correll, 2014; Bozzatello et al., 2019); since our population included young patients, age of onset was mirrored by age at the evaluation. Moreover, older patients have on the average a longer disease duration and received many different medications. This is relevant since the probability of response to antipsychotics is higher at the first episode of psychosis, and decreases after multiple acute episodes (Haddad and Correll, 2018). Then, changes in biochemistry, functional connectivity, vasculature, synapsis remodeling and neuronal death occurs during brain aging and maturation, possibly provoking differences in AP pharmacodynamics (Shi and Klotz, 2011) Last, changes in pharmacokinetics related to age can influence the bioavailability of AP in the brain and the other bodily compartments^63^. However, our target was to realize a real-world study including heterogeneous patients, in order to have results to extend to all subjects prescribed with antipsychotics. In order to control the possibly confounding effect of age, it was included in the regression analysis model.

In conclusion, the present study shows that genetic factors previously associated in GWAS studies to response to antipsychotics but not to schizophrenia are associated with response to treatment in schizophrenic patients from a naturalistic cohort study. On the other hand, this result cannot be translated into a clinical tool to predict response.

## Data Availability

Due to the sensitivity of the data and the absence of informed consent for public data depository, the datasets analyzed during this study are not available to the public. The dataset supporting the conclusions of this article were obtained from PsyMetab study. Requests to access the datasets should be directed to: research.psymetab@chuv.ch.
